# Use of Sysmex XN‐10 red blood cell parameters for screening of hereditary red blood cell diseases and iron deficiency anaemia

**DOI:** 10.1111/ijlh.13278

**Published:** 2020-07-08

**Authors:** Vanessa Nivaggioni, Lakhdar Bouriche, Sylvie Coito, Anne‐Sophie Le Floch, Manal Ibrahim‐Kosta, Caroline Leonnet, Isabelle Arnoux, Marie Loosveld

**Affiliations:** ^1^ Laboratoire d'Hématologie Hôpital de la Timone Assistance Publique – Hôpitaux de Marseille Marseille France; ^2^ Laboratoire SYNLAB Provence Marseille France; ^3^ Laboratoire Ketterthill Belvaux Luxembourg; ^4^ Aix Marseille Univ CNRS INSERM CIML Marseille France

**Keywords:** Iron deficiency anaemia, laboratory practice, RBC and reticulocyte parameters, RBC disease, Sysmex XN‐10

## Abstract

**Introduction:**

In daily practice in haematology laboratories, red blood cell (RBC) abnormalities are frequent and their management is a real challenge. The aim of this study is to establish a “decision tree” using RBC and reticulocyte parameters from the SYSMEX XN‐10 analyser to distinguish between patients with a hereditary RBC disease from iron deficiency anaemia and other patients.

**Methods:**

We analysed results of complete RBC counts in a cohort composed of 8217 adults divided into 5 different groups: iron deficiency anaemia (n = 120), heterozygous haemoglobinopathy (n = 92), sickle cell disease syndrome (n = 56), hereditary spherocytosis (n = 18) and other patients (n = 7931). A Classification And Regression Tree (CART) analysis was used to obtain a two‐step decision tree in order to predict these previous groups.

**Results:**

Five parameters and the calculated RBC score were selected by the CART method: mean corpuscular haemoglobin concentration, percentage of microcytes, distribution width of the RBC histogram, percentage of nucleated red blood cells, immature reticulocytes fraction and finally RBC Score. When applying the tree and recommended flowchart, 158/166 of the RBC hereditary disease patients and 114/120 iron deficiency anaemia patients are detected. Overall, the correct classification rate reached 99.4%. Sensitivity and specificity for RBC disease detection were 95.2% and 99.9%, respectively. These results were confirmed in an independent validation cohort.

**Conclusion:**

Based on the XN‐10 RBC and reticulocyte parameters, we propose a two‐step decision tree delivering a good prediction and classification of hereditary RBC diseases. These results can be used to optimize additional reticulocyte analysis and microscopy review.

## 1 INTRODUCTION

According to the World Health Organization (WHO), anaemia is a global public health problem affecting 1.6 billion people around the world, which corresponds to approximately 25% of the world population.^1^ Different aetiologies lead to anaemia or red blood cell (RBC) abnormalities. Iron deficiency anaemia (IDA) is considered to be the main cause of anaemia. Indeed, the proportion of anaemia associated with iron deficiency was 25% for preschool children and 37% for nonpregnant women of reproductive age.^2^ Besides iron deficiency, hereditary red blood cell diseases including haemoglobinopathy, red cell membrane pathology and enzymatic deficiency are also the common origin of RBC parameter disorders. The current estimation of individuals carrying a significant variant of haemoglobin is up to 323 million (5.2%) in the world.^3^ The estimated number of births with a serious inherited haemoglobin disorder is still up to 330 000 per year (83% sickle cell disease, 17% thalassaemias) and contributes to the equivalent of 3.4% of mortality in children aged under 5 years worldwide or 6.4% in Africa.

In daily laboratory practice, the use of automated haematology analysers brings the reliability of the results but no analyser can determine properly RBC morphological abnormalities and cytomorphological examination of the blood smear is necessary to interpret automated results for unknown patients. The decision of blood smear examination is triggered by one or more alarms generated by the analyser or quantitative and/or qualitative criteria decided by the laboratory performing the analysis. These criteria are not always relevant hence the interest to associate several RBC and reticulocyte parameters in order to improve the specificity and sensitivity of microscopy examination of a blood smear.

Different studies have been already described for discriminating between iron deficiency anaemia and thalassaemia trait, but none of them take into account more clinical situations including both constitutional and acquired anaemia. The aim of this study was to establish a decision tree using RBC and reticulocyte parameters from the SYSMEX XN‐10 to distinguish between patients with hereditary RBC disease from iron deficiency anaemia and other patients. Another goal was to focus on the patients flagged for RBC disease in order to specify RBC pathologies.

## 2 MATERIALS, METHODS AND PATIENTS

### 2.1 Materials and Methods

The study was approved by the local Ethics Committee of Assistance Publique des Hôpitaux de Marseille (Marseille, France).

Blood samples were collected in EDTA K3 Becton Dickinson Vacutainer^TM^ tubes (Franklin Lakes, NJ, USA) and analysed within 6 hours after collection.

Complete blood counts were performed using Sysmex XN‐10 (Sysmex Corporation^TM^, Kobe, Japan) analysers equipped with a reticulocyte analysis module.

The RBC count was measured using the impedance variation method after hydrodynamic focusing. The haematocrit (HCT) was obtained from the cumulative values of the individual cell pulse heights. The haemoglobin (HGB) concentration was measured by photometry with the reagent sodium lauryl sulphate. Mean corpuscular volume (MCV), mean corpuscular haemoglobin (MCH) and mean corpuscular haemoglobin concentration (MCHC) were calculated from RBC, HGB and HCT using the classical formula. The Micro% is an additional RBC parameter indicating the percentage of microcytic RBC with a volume of less than 70 fL. A red blood cell distribution width called RDW‐SD is also available; RDW‐SD corresponds to the curve distribution width at 20% of the height of the peak.

Samples presenting with an increased MCHC value (>365 g/L) were reflexed in the RET (reticulocyte) channel and classified as per the CBC‐O application^4^ developed by Sysmex that is embedded in the *Extended* Information Processing Unit (*Extended‐*IPU). The RBC score was calculated as per Berda et al recommendation and embedded reticulocytes absolute count and the percentage of fragments reported by the XN analyser when reticulocytes are measured.

Flow cytometry provides the leucocyte count and identifies erythroblasts (NRBC%) via the WNR (white and nucleated red blood cells) channel and counts reticulocytes via the RET channel. The IRF% parameter defines the immature reticulocyte fraction.

XN‐10 RET is used in daily routine in the laboratory, adjustments and calibrations are checked regularly, and controls are analysed each day using internal quality control from Sysmex ^TM^.

### 2.2 Patients

8217 adults, 15 years of age and older, were analysed in a retrospective study. The first haemogram of 8122 patients was randomly collected over a four‐week period in February 2019 and enriched with 95 supplementary patients in order to increase the number of RBC diseases and iron deficiency anaemia. These additional patients were collected over a three‐year period (2014‐2016).

All patients were issued from the Marseille University Hospital (France) and were divided into 5 different groups: iron deficiency anaemia “IDA,” heterozygous haemoglobinopathy “HGB HTZ,” sickle cell disease “SCD,” hereditary spherocytosis “HS” and other patients who did not have a RBC disease or iron deficiency anaemia “OTHERS.”

Diagnosis of IDA was based on ferritin made immunoturbidimetrically via Cobas 8000 (c701 module) Roche Diagnostics with a cut‐off value < 15 µg/L for patients with MCV under normal reference values.^5^ High‐pressure liquid chromatography on a Bio‐Rad Variant™ II analyser, capillary electrophoresis on SEBIA® Capillarys 2 and molecular diagnosis if necessary were used to make diagnosis of haemoglobinopathy. Diagnosis of HS was obtained with a positive eosin‐5‐maleimide binding test (EMA test) according to clinical and biological history.

In the group “OTHERS,” patients fitting the normal reference values have not been further investigated. For patients with at least one abnormality in the RBC parameter panel, we sampled 100 of them and checked their CBC indication. No IDA or RBC disease was found. These results were extrapolated to the rest of this subgroup. Consequently, all these mentioned tests (Ferritin, EMA test..) were not performed for all patients but only according to the call points and the context. If data were not available or if patient were receiving therapy affecting erythropoiesis (Iron therapy, blood transfusion, EPO), they have been excluded from the cohort.

### 2.3 Validation cohort

The decision tree was subsequently tested in an independent validation cohort of 14 705 patients (>15 years old) from private Laboratory Ketterthill (Esch SUR/Azeltte, Luxembourg) with a recruitment exclusively issued from general practician collected over a two‐month period between 1st of July and 3rd of September 2019. Patients were divided into the same five groups, and diagnosis was obtained via similar reference methods. The research for validation cohort was exempted of IRB approval.

### 2.4 Statistical Evaluation

RBC parameters were analysed as quantitative variables and summarized as median and range.

The association between RBC and reticulocyte parameters and groups was performed using the Kruskal‐Wallis test with Dunn's multiple comparison test.

A Classification and regression tree (CART) analysis was performed to identify predictive RBC and reticulocyte parameters for the different groups (iron deficiency anaemia, heterozygous haemoglobinopathy, sickle cell disease, hereditary spherocytosis and other patients). Internal validation was performed using 10‐fold cross‐validation. The choice of variables and their thresholds is inherent to the CART methodology.

All tests were two‐sided at a 0.05 significance level. All analyses were performed using R statistical software V.3.3.2 (R Foundation for Statistical Computing) and XLSTAT 2019 from Microsoft Corporation ^TM^.

## 3 RESULTS

### 3.1 Patients

8217 patients were divided into groups as follow: “IDA” (n = 120, 1.5%, mean ferritin level = 8.0 µg/L), “HGB HTZ” (n = 92, 1.1% ‐ β‐thalassaemia trait [n = 83] ‐ α‐thalassaemia trait [n = 3] ‐ HbS trait [n = 3] ‐ HbC trait [n = 2] HbE trait [n = 1]), “SCD” (n = 56, 0.7% ‐ S/S disease [n = 39], sickle cell thalassaemia [n = 14] and S/C disease [n = 3]), “HS” (n = 18, 0.2%) and “OTHERS” (n = 7931, 96.5%).

In the group called “OTHERS”, 4008 patients were fitting the normal reference values,^5^ while 3923 presented with at least one abnormality from the panel of RBC parameters (Table S1). All patients were older than 15 years, and the sex ratio was between 0.60 and 1.04 depending on the groups.

### 3.2 FIRST STEP: Screening of RBC disease and IDA

#### 3.2.1 Red blood cell parameters

**Figure 1:**
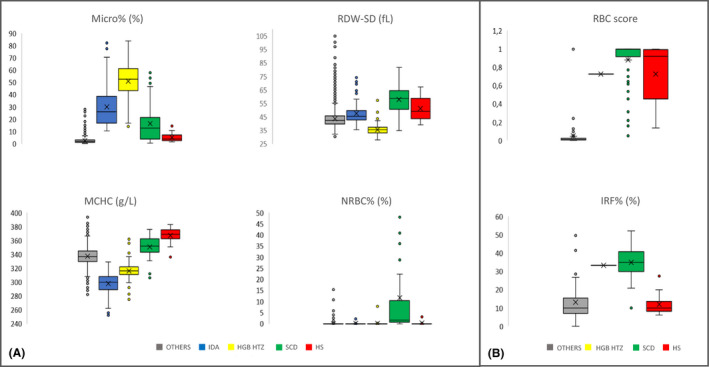
Group comparison with Kruskal‐Wallis test between five groups (others patients (grey), iron deficiency anaemia (blue), heterozygous haemoglobinopathy (yellow), sickle cell disease (green), hereditary spherocytosis (red) – (A) for Micro%, RDW‐SD, MCHC and NRBC% for all the cohort [step 1] – (B) for RBC score and IRF% for patients presenting with MCHC > 365 g/L and for patient who need the addition of RET channel in step 1 [step 2]

Results of the complete RBC count were analysed in this cohort and summarized in Table 1. Graphical representations of four main parameters proposed by the CART method are shown (Figure 1A).

**Table 1:** Median, minimum and maximum values for analysed RBC parameters in the 5 groups

	Others	Iron deficiency anaemia	Heterozygous haemoglobinopathy	Sickle cell disease	Hereditary spherocytosis
N	7931	120	92	56	18
Sexe ratio (M/F)	3994/3937 (1.01)	47/75 (0.60)	47/45 (1.04)	21/35 (0.60)	8/10 (0.8)
Age (years)	56 (15‐102)	53 (15‐97)	52 (15‐91)	29 (15‐56)	29 (15‐62)
RBC (T/L)	4.54 (1.41‐7.35)	4.54 (1.84‐7.38)	5.96 (3.77‐7.27)	3.01 (1.31‐4.97)	4.01 (2.60‐4.95)
MCV (fL)	87.9 (69.0‐121.8)	72.2 (51.4‐83.0)	63.0 (50.5‐74.1)	82.5 (60.5‐115.5)	86.7 (79.5‐98.1)
Micro% (%)	2.0 (0.1‐28.2)	26.0 (10.6‐82.0)	52.7 (14.3‐83.7)	12.8 (0.7‐57.9)	4.0 (1.7‐14.4)
RDW‐CV (%)	13.2 (10.7‐30.3)	18.6 (14.5‐32.3)	17.6 (13.6‐23.8)	19.9 (14.0‐27.9)	15.9 (13.4‐22.0)
RDW‐SD (fL)	42.5 (30.5‐126.3)	45.6 (35.4‐74.3)	35.5 (28.1‐57.1)	58.9 (35.0‐81.7)	49.2 (39.2‐67.3)
HGB (g/L)	134 (48‐207)	92 (34‐182)	117 (81‐147)	91 (52‐126)	130 (88‐158)
MCH (pg)	29.7 (21.4‐42.0)	21.8 (13.9‐25.2)	19.9 (15.1‐26.3)	29.3 (20.7‐41.7)	31.7 (29.4‐35.4)
MCHC (g/L)	337 (282‐394)	300 (252‐329)	316 (275‐362)	352 (306‐376)	369 (336‐383)
NRBC% (%)	0.0 (0.0‐15.3)	0.0 (0.0‐2.1)	0.0 (0.0‐7.8)	1.7 (0.0‐199.3)	0.0 (0.0‐3.0)

In the group “OTHERS,” the median value of percentage of microcytes (Micro%), mean corpuscular haemoglobin concentration (MCHC), red blood cell distribution width (RDW‐SD) and nucleated red blood cells (NRBC%) were, respectively, 2.0% [0.1‐28.2], 337g/L [282‐394], 42.4fL [30.5‐126.3] and 0.0% [0.0‐15.3]. These values were significantly different from the four other groups (*P* ≤ .001).

“IDA” and “HGB HTZ” patients had an increased Micro% (26.0% [10.6‐82.0] and 52.7% [14.3‐83.7]), significantly different from other groups (*P* ≤ .001) and a quite low MCHC: 300g/L [252‐329] and 316g/L [283‐362], respectively (*P* = .014). RDW‐SD differed drastically between “IDA” (45.6fL [35.4‐74.3]) and “HGB HTZ” patients (35.5fL [28.1‐57.1]), *P* < .0001. In the group IDA, sex was not distinguished since a preliminary comparison between male and female did not demonstrate any significant difference in all studied parameters.

Micro% was not significantly different between “SCD” and “HS” patients (12.8% [0.7‐57.9] and 4.0% [1.7‐14.4], *P* = .371) as MCHC (352g/L [306‐376] and 369g/L [336‐383], *P* = .027). However, MCHC in these two groups was significantly different from the rest of the cohort (*P* < .001).

The presence of nucleated red blood cells statistically differentiated the “SCD” group (1.7% [0.0‐199.3]) from the rest of the patients (*P* < .0001).

#### 3.2.2 CART tree RBC

Out of 8217 patients, 82 presented with an increased MCHC > 365g/L. These patients were not analysed in the RBC tree but reserved for the RET channel as per Berda‘s recommendations and classified separately at the RBC step. Patients presenting with cold agglutinins were excluded due to the inability to interpret the Micro% issued by the analyser. Patients presenting with optical interference were previously corrected by the use of the CBC‐O application and then integrated into the CART tree RBC. Patients with increased MCHC appeared here highly heterogeneous with the inclusion of 12 patients with SCD, 12 with HS and 58 patients issued from the “OTHERS” population.

**Figure 2:**
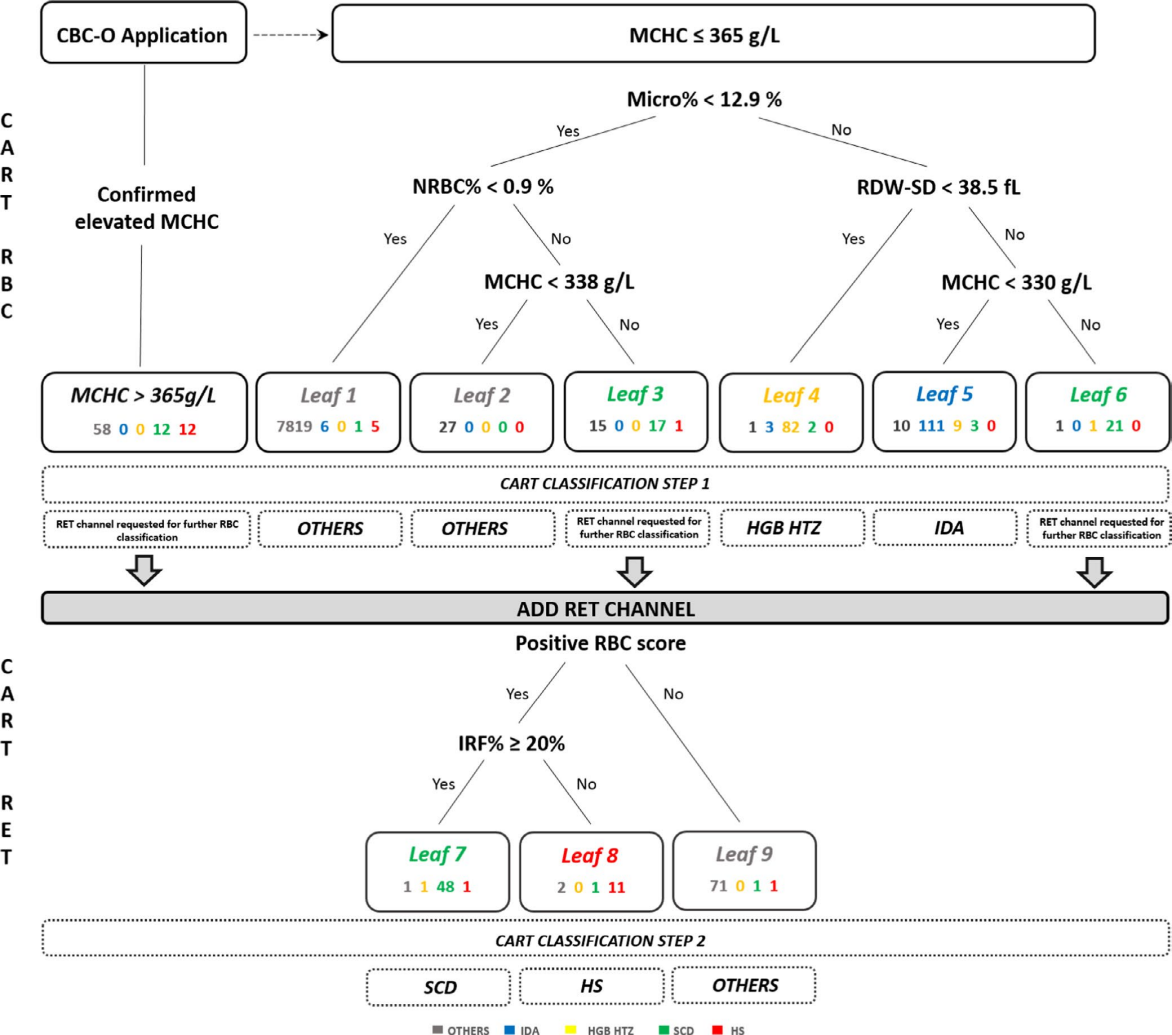
Global decision tree included CART CBC (step 1) and CART RET (step 2). “OTHERS” in grey, “IDA” in blue, “HGB HTZ” in yellow, "SCD" in green, “HS” in red

All 8135 remaining patients were distributed into 6 leaves. As described above, four parameters and their cut‐off values were selected by the CART method for the decision tree based on patients with a normal MCHC (≤ 365 g/L): Micro% (%), NRBC% (%), RDW‐SD (fL) and MCHC (g/L). The CART RBC tree is shown in Figure 2.

In leaf 1 and leaf 2, 7846/7858 patients belonged to the “OTHERS” group. Six mild IDA (MCV between 77.9 and 83.0 fL) were present, one SCD and 5 HS were also found. Regarding these 5 HS patients, the mean HBG was 127 g/l (92‐153) and the mean MCHC was 356 g/l (336‐365).

Leaf 4 included 82 patients with a heterozygous haemoglobinopathy. Three IDA and 2 SCD presenting with a narrow RBC distribution curve (RDW‐SD < 38.5 fL) were observed. One patient from “OTHERS” was included for the same reason (RDW‐SD 36.4 fl).

Among 133 patients, leaf 5 included 111 IDA patients and 12 RBC disease patients (2 sickle cell thalassaemia, 1 S/S disease, 5 β‐thalassaemia trait, 1 HbE trait, 1 HbS trait and 2 α‐thalassaemia trait) demonstrated a normal ferritin level. The remaining 10 patients had an inflammatory anaemia (n = 9) and schistocytes (n = 1).

Leaves 3 and 6 were mainly composed of SCD patients, but there were also 16 patients belonging to the group “OTHERS” and two additional RBC disease patients (1 HS and 1 HGB HTZ).

Overall, leaves 1 and 2 predicted “OTHERS”, leaf 4 “HGB HTZ” and leaf 5 “IDA” groups. A good classification rate for “IDA,” “HGB HTZ” and “OTHERS” patients without an elevated MCHC reached 92.5% (111/120), 89.1% (82/92) and 99.7% (7846/7873), respectively. Moreover, only one sickle cell disease and 5 hereditary spherocytosis were misclassified in leaf 1 and 12 RBC disease samples were classified as IDA (leaf 5).

### 3.3 SECOND STEP: specification of RBC disease

#### 3.3.1 Reticulocyte parameters

In this second step, RET analysis was added for patients who were predicted in leaves 3 and 6 by CART RBC (n = 56, 0.7% of global cohort) as it was automatically done for patients with MCHC > 365 g/L (n = 82). Graphical representations of the two selected parameters are shown (Figure 1B, n = 138).

The RBC score involving RET and FRC^4^ provided an excellent differentiation between other patients and RBC disease patients (*P* < .0001).

Obviously, the reticulocyte count was elevated in both “HS” and “SCD” but IRF% remained very different, 34.8% [10.0‐52.5] for SCD patients versus 12.3% [6.1‐27.5] for spherocytosis (*P* < .0001) (data not shown).

#### 3.3.2 CART tree RET

Two parameters were selected for the second decision tree: RBC score and IRF%. RBC score and its cut‐off value (0.15) are part of the CBC‐O application and cannot be changed. The second parameter was selected by the CART method. All 138 patients were distributed into 3 leaves (Figure 2).

In leaf 7, 48 patients belonged to the SCD group. One HGB HTZ and one HS with an extremely high reticulocyte count (783 G/L) were present. One patient from “OTHERS” was as well found. This patient was suffering from an autoimmune haemolytic anaemia.

Leaf 8 was mainly composed of HS patients (11/14). Additionally, one SCD and two “OTHERS” were present without any special observation.

Among 73 patients classified in this leaf 9, 71 were confirmed as “OTHERS.” One SCD and one HS were also found. Both of them presented with a normal reticulocyte count and absence of FRC%, leading to a normal RBC score.

Overall, two leaves predicted RBC disease, SCD in leaf 7 and HS in leaf 8. Leaf 9 removed RBC disease false positives from the CART RBC.

In conclusion (Table 2), the good classification rate reached 99.4%. Out of the misclassified patients, only two SCD and six HS were found false negative, 12 RBC diseases were predicted in IDA and five RBC diseases were not in the right RBC disease classification. Four “OTHERS” patients were RBC disease false positive and good classification for this group reached 99.8% (7917/7931). Sensitivity and specificity to predict RBC disease were 95.2% [90.6%‐97.7%] and 99.9% [99. 8%‐100.0%], respectively, with a 95% confidence interval.

**Table 2:**
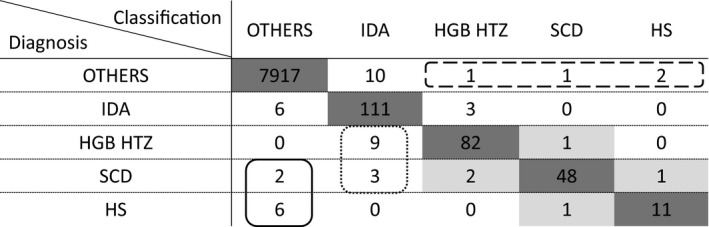
Summary of CART tree classification. Good classification in dark grey, IDA false positive in dotted line, RBC disease false negative in plain line, RBC disease false positive in dashed line and RBC disease true positive with wrong classification in light grey

#### 3.3.3 Validation cohort

The validation cohort included 14 705 patients divided into the same five groups as follow: “IDA” (n = 111, 0.76%), “HGB HTZ” (n = 71, 0.5% ‐ β‐thalassaemia trait [n = 55] ‐ α‐thalassaemia trait [n = 6] ‐ HbS trait [n = 8] ‐ HbC trait [n = 1] ‐ HbE trait [n = 1]), “SCD” (n = 3, 0.02% ‐ S/S disease [n = 1], sickle cell thalassaemia [n = 2]), “HS” (n = 3, 0.02%) and “OTHERS” (n = 14 517, 98.7%). Applying the established CART in this cohort, only 8 RBC diseases were found false negative. Sensitivity and specificity to predict RBC disease reached 89.6% [80.5%‐94.8%] and 100.0% [99.9%‐100.0%], respectively, with a 95% confidence interval. 93/111 iron deficiency anaemia patients are detected. To note, out of 2252 ferritin analysed, 336 patients without microcytic anaemia have a ferritin level < 15 µg/L in this cohort (or 2.0% of the group OTHERS). Overall, the good classification rate reached 99.7%.

## 4 DISCUSSION

The aim of our study was to establish a decision tree using RBC and reticulocyte parameters from the XN‐10 analyser providing the RBC disease detection. Indeed, previous studies have demonstrated the interest of mathematical indices to discriminate RBC diseases in the context of microcytic anaemia.^6^ However, these studies mostly focus only on IDA and β‐thalassaemia trait in subjects with microcytic RBC and additionally, none of them consider the situation where patients are not presenting with anaemia. Moreover, none of these indices are able to reach absolute sensitivity and specificity. In 2015, Hoffmann et al^6^ undertook the first meta‐analysis of the 12 most frequently used discriminant indices. Despite high variation in the performance of these indices, they have demonstrated the superiority of the M/H ratio^7^ over other discriminant indices. In our decision tree, statistical robustness of Micro% is used to directly distinguish between IDA, RBC disease and other causes of RBC abnormality instead of MCV and MCH, two traditional parameters used for anaemia classification. Because of the heterogeneity of our population, Micro% was not sufficient alone to predict an outcome. Other parameters are also required. RDW‐SD measures anisocytosis and helps to differentiate thalassaemia trait (without anisocytosis) from IDA and SCD with Micro%> 12.9%. NRBC% is typical of SCD, and MCHC helps for HS, SCD (elevated MCHC), IDA and HGB HTZ (low MCHC).

**Figure 3:**
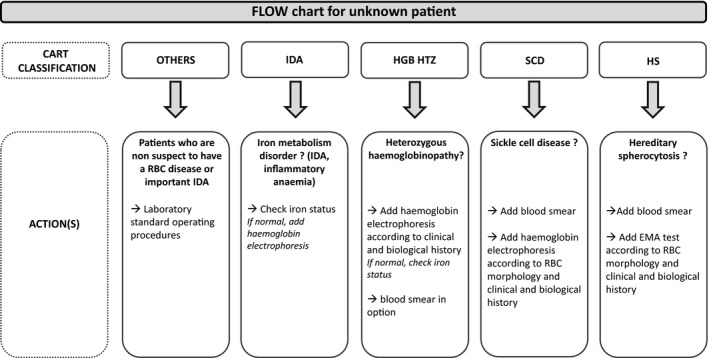
Flowchart for unknown patient

This first step is sufficient to well classify 92.5% of “IDA,” 89,1% of “HGB HTZ” and 99.7% of “OTHERS.” Nevertheless, few patients were misclassified: some “IDA” in OTHERS (6 /7831 in leaf 1) and some inflammatory anaemia (9 “OTHERS”) or RBC disease patients (9 “HGB HTZ” and 3 “SCD”) in leaf 5. To avoid these misclassified patients, first we advise to check iron status and if iron status is normal to add haemoglobin electrophoresis in a second step (Figure 3). More, heterozygous haemoglobinopathy was well predicted by the CART CBC (leaf 4). Most of the patients in this group had a β‐thalassaemia trait. Four of the misclassified “HGB HTZ” patients had no β‐thalassaemia trait but HbE, HbS or α‐thalassaemia trait. Hence, the poorer results of the HBG HTZ group (89.1%) can be partly explained by the heterogeneity of the patients included. To avoid these misclassified patients, we advise to add iron status if haemoglobin electrophoresis is normal (Figure 3). It is important to note here that this algorithm does not allow to predict low ferritin levels without RBC parameter abnormalities but permits to distinguish heterozygous haemoglobin from iron deficiency.

The second step of our decision tree requires reticulocyte parameters. Indeed, some of the SCD and HS patients had an increased MCHC > 365 g/L and were managed by the CBC‐O application. The remaining SCD and HS patients were predicted by the CART analysis in two different leaves (3 and 6). In these cases, adding RET channel (+ 0.7% reticulocyte count) is also requested. The addition of reticulocyte parameters allowed to properly file some RBC disease false positives thanks to the RBC score (71 in our cohort). Moreover, the adding of IRF% gives a clear differentiation between the two RBC diseases with high reticulocyte count (SCD and HS). Indeed, IRF% is an excellent parameter to screen HS as previously described by Mullier et al on XE‐series^8^ and recently validated by Sottiaux et al on XN‐Series.^9^ This second step is necessary to reach a good sensitivity and specificity for RBC disease detection, respectively, of 95.2% and 99.9% and that is why we advise to do a blood smear review and, according to RBC morphology and clinical and biological history, to add further additional laboratory investigations (haemoglobin electrophoresis or EMA test,^10^ Figure 3).

Of course, some limitations of our study can be underlined. Our algorithm is only applicable to patients whose blood count has been performed on the Sysmex XN‐10 series because some specific parameters are included in this tree therefore not transposable to another type of automaton. It should also be noted that this study is designed to isolate RBC disease versus IDA when present rather than giving a focus on discrimination between heterozygous haemoglobinopathy and IDA in the limited context of microcytic anaemia. As such, this tree is not supposed to detect all patients presenting with iron deficiency.

Overall, these two‐step decision tree allow us to reach a very good classification rate (99,4%) and these results were also confirmed by an external cohort. Our study takes into account many clinical situations and represents a daily university laboratory routine practice. This algorithm is able to well differentiate several causes of anaemia, both acquired and constitutional, and not only IDA or thalassaemia. Only few “other” patients or iron deficiency anaemia interfere with the RBC disease detection. Up until now, few investigators have also included subjects with other types of haemoglobinopathy, such as HbE^11^ and HbS, both sickle cell thalassaemia and sickle cell diseases^12^ or inflammatory anaemia.

## 5 CONCLUSION

Based on the XN‐10 RBC and reticulocyte parameters, we propose for Sysmex users a decision tree and a flowchart that delivers an interesting prediction of RBC hereditary disease while considering iron deficiency anaemia as a preliminary step to further investigation.

## AUTHORS’ CONTRIBUTION

All authors participated substantially so as to be considered authors in this manuscript. All authors read and approved the final manuscript. V. Nivaggioni designed the research study. V. Nivaggioni, A‐S. Le Floch, L. Bouriche and Manal Ibrahim performed the research and analysed the data. Sylvie Coito proceeded with the validation cohort. V. Nivaggioni, L. Bouriche and M. Loosveld wrote the paper.

## Supporting information

Table S1
