# Supplementary figures and images for: Use of Sysmex XN‐10 red blood cell parameters for screening of hereditary red blood cell diseases and iron deficiency anaemia
Source: Int J Lab Hematol. 2020 Jul 8;42(6):697–704. doi: 10.1111/ijlh.13278 (PMC7754411; doi:10.1111/ijlh.13278)

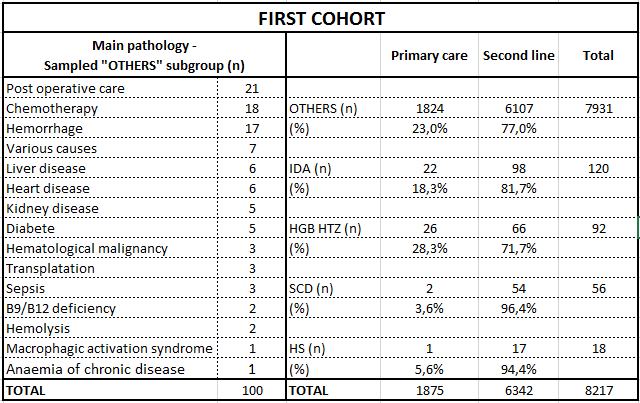

Supplement: Supplementary file 1 — Table S1 [file IJLH-42-697-s001.png]
